# The Sound of Grief: A Critical Discussion on the Experience of Creating and Listening to the Digitally Reproduced Voice of the Deceived

**DOI:** 10.1177/00302228231225273

**Published:** 2024-01-04

**Authors:** Silvia Francesca Maria Pizzoli, Laura Vergani, Dario Monzani, Ludovica Scotto, Clizia Cincidda, Gabriella Pravettoni

**Affiliations:** 1 Department of Oncology and Hemato-Oncology, 9304 University of Milan, Milan, Italy; 2 Department of Psychology, Univeristà Cattolica Del Sacro Cuore di Milano, Milan, Italy; 3 Department of Psychology, Educational Science and Human Movement, 18998 University of Palermo, Palermo, Italy; 4 Applied Research Division for Cognitive and Psychological Science, 9290 IEO European Institute of Oncology IRCCS, Milan, Italy

**Keywords:** grief, bereavement, artificial intelligence, audio clip

## Abstract

Technological tools allow for the reproduction and control of peculiar stimuli, such as the possibility of producing audio clips with the voices of deceased people. Artificial intelligence allows to create at-home vocal messages from an audioclip. Recently, some videos and documentaries depicting people interacting with artificial intelligence content related to the deceased have been released to the general public. However, the possibility of interacting with realistic stimuli related to deceased loved ones can create peculiar and delicate experiences and should gain the attention of the scientific community and mental health professionals. Listening and searching for experiences related to the deceived ones might indicate a natural way to elaborate and live the experience of grieving or the presence of symptoms related to more severe conditions. Moreover, such powerful stimuli might be potentially harmful to users, if not appropriately used. To the best of our knowledge, no scientific literature exists on the topic of listening to audio clips with the voice of the deceased yet, although various people shared thoughts and feelings about these habits on social networks and forums. Given the relevant psychological impact that grief can have on a person, an open discussion on the possibility and risks of the availability of digital stimuli related to grief should be taken into account by the scientific community.

“Because loss is forever, so too is the state of being bereaved” (Simon, 2013)

## Introduction

### Grief and Digital Stimuli

In recent years, technological tools able to offer realistic illusions to the users’ senses and minds have dramatically increased and become more easily available. It is the case of virtual reality (VR) devices or software based on artificial intelligence (AI) algorithms, which can create ‘impossible’ yet realistic stimuli. From our homes, we can use virtual glasses or headsets to see virtual environments with dinosaurs or talk to chatbots that mimic natural conversations while using our smartphones.

The present work focuses in particular on the possibility of creating audio clips, multimedia messages, and even conversations featuring voices and semblances of deceased people.

It is the case of AI-generated podcasts, broadcasting fictional conversations and interviews of famous, deceased, people; also, there is the possibility of selecting pre-recorded videos to create artificial interactions with dead persons. It is the case of an 87-year-old woman, whose image and voice were reproduced on a screen during her funeral with AI techniques programmed by her son and his wife. Specifically, the reproduced video of the dead woman was programmed to answer real-time questions from the familiars and beloved ones, taking information and answers from a store of previously pre-recorded video clips of the woman (“AI lets deceased woman address her funeral | Artificial Intelligence tool helps the dead talk | WION - YouTube,” 2023) Recently, as announced during a conference, engineers of an Amazon company are working on an already-existent and commercially-available intelligent personal assistant to enrich it with the capability of mimicking the voice of a deceased person; this will be possible using only less than a minute vocal recording of the departed person’s voice. In the company demonstration, the personal assistant starts reading a kid a tale, mimicking the voice of his grandmother. No further details have been shared at the moment, but we may suppose that this could potentially be a technology available to a vast audience of people, whenever they want, from the comfort of their homes. (“Amazon’s Alexa could turn dead loved ones’ voices into digital assistant | Amazon Alexa | The Guardian,” 2023)

The fact that digital stimuli related to deceased people might be available to a broad audience should be carefully discussed.

## Results

### Complicated Bereavement and the Psychological Impact of Digital Stimuli Related to Grief

From a psychological perspective, the psychological impact and risk of these experiences, which might be relevant, are still unclear.

A first consideration might be done on why people should choose to interact with digitally reproduced features of a dead person.

In grieving people, listening and searching for experiences related to the deceived ones might indicate a natural way to elaborate and live the experience of grieving. In other cases, searching for things associated with the lost person might indicate the presence of symptoms related to more severe conditions, such as a persistent complex bereavement disorder (American Psychiatric Association, 2022) or prolonged grief disorder (PGD) (Killikelly & Maercker, 2017), that occurs in about 7% of the grieving person (Kersting et al., 2011), cause functional impairments and are associated with suicidal thoughts (Shear & Kenworthy, 2012) Indeed, among the possible signs of bereavement issues, there is “excessive proximity seeking, eg, refraining from going places, doing things, or having contact with things that are reminders of the loss, or feeling drawn to reminders of the person, such as wanting to see, touch, hear, or smell things to feel close to the person who died” (Shear et al., 2013). Unfortunately, it is difficult to recognize the signs and patterns of complicated grief and to distinguish the disorder from the natural process of grieving. The diagnosis of disorders of grieving can be difficult since the trajectory of bereavement is complicated to be predicted and it might be ongoing for a long period (Kersting et al., 2011). Plus, the very same person might behave in conflicting ways and contemporary avoid reminders of the loss, and spend energy trying to feel close to the dead person (Shear & Kenworthy, 2012).

The mourning experience, by exposing ourselves to a loss, activates the attachment mechanism (Sekowski & Prigerson, 2022; Shear & Shair, 2005). While a link between disorganized attachment and PGD has been established (Sekowski & Prigerson, 2022), also for people with an ambivalent-insecure attachment style, the availability to reuptake the image, the voice, and the main characteristics of the lost beloved could represent a psychological threat. Indeed, the possibility to program an ad hoc conversation may reinforce the tendency to deny painful yet true cognitions of the loss. Moreover, the videos or the audio clips might act like a compensation mechanism that offers momentary consolation, but they do not permit the physiological flow of the lost pattern.

The issue of clinical syndromes associated with grief increased during the pandemic, when the PGD became a major matter (Eisma et al., 2020), due to social distancing and difficulties to reach psychological support.

### How People Might Interact With Digital Stimuli Related to Grief?

While - to the best of our knowledge - no scientific literature exists on the topic of listening to audio clips with the voice of the deceased yet, various people shared thoughts and feelings about these habits on social networks and forums, for example Reddit (source: Reddit). People reported listening to voicemail messages of their beloved departed ones, with various frequencies and effects: some find the experience to be too painful, others to be comforting. This variety of effects reflects the complex experience of bereavement, different from person to person (Parkes & Prigerson, 2013). We might also conclude that the need for listening to the voice of departed beloved people while grieving is not rare.

However, the difference between listening to already registered audio clips and interacting with AI content is not trivial. The first is the case of audio clips really registered by the departed ones, with their real voices; for example, a voicemail that a grandfather himself left on a niece’s phone some days before his death, wishing her “happy birthday” or whatever he wanted to say to her. The latter is the case of ad hoc created stimuli, mimicking real voices and semblances of deceased people; for example, an AI-generated video message of a departed aunt, some years after she passed away, featuring content decided by the nephew or by some other person or technology.

In a recent work, van Minnen and colleagues (van Minnen et al., 2022) used AI and deep fake technology with ad hoc stimuli for the treatment of two women who had been sexually assaulted. The participants were suffering from post-traumatic stress disorder because of the sexual trauma and underwent a session of confrontation with the perpetrators, reproduced thanks to the deep fake technique. Both the participants, which were completely aware of the nature of the stimulus, reported benefits after the confrontation.

Starting from the documentary “*I met you*”, which reported the meeting between a mother and her virtually reproduced seven-year-old daughter who recently passed away, it is important to point out the possibility that such powerful stimuli might be potentially harmful to users, if not appropriately used (Pizzoli et al., 2021). The work also tried to highlight potential benefits and risks related to the development of clinical intervention employing the use of realistic content related to grief. Specifically, the authors called for a systematic process of steps of empirical validation of virtual intervention and stimuli related to grief (Birckhead et al., 2019).

In the present work, we are referring to slightly different technologies, which can give rise to distinct psychological experiences compared to VR. Indeed, the VR can create a vivid and realistic illusion of an environment and avatars and the users might behave and feel their emotions as if they really were inside the VR (Slater, 2018). However, VR creates the perception of an alternative reality that does not coincide or completely overlap with the physical one. VR requires specific hardware tools to create the illusion of the stimuli (glasses, specific rooms, and so on) and the stimuli themselves are realistic, yet digital images that can be easily identified as digital percepts. Furthermore, when avatars are employed, the perception of the faces or humanoid bodies can elicit uncanny or oddly familiar feelings of uneasiness and revulsion in users (Kätsyri et al., 2015; Mori et al., 2012). In the case of audio clips or videos reproduced with AI algorithms, it is still unclear if there is at least a certain degree of perceptual awareness that the stimulus has been digitally created. Kobis and colleagues (Köbis et al., 2021) found that people’s ability to detect deepfake videos is generally low, with a bias “toward mistaking deepfakes as authentic videos (rather than vice versa)” and an overestimation of their abilities. To the best of our knowledge, no experiment has ever been conducted using in particular stimuli related to dead people. Furthermore, VR environments are usually pre-programmed and offer “fixed” solutions to the users. While it is unlikely that users will program a home-based virtual scenario with elements related to grief, it is less improbable to train commercially available intelligent personal assistants to recreate the voice of the dead.

## Discussion and Future Directions for Health Professionals and the Scientific Community

Given the relevant psychological impact that grief can have on a person, an open discussion on the possibility and risks of the availability of digital stimuli related to grief should be taken into account by the scientific community.

Firstly, considering that the search for experiences related to deaths is not rare in both natural and complicated bereavement, an extensive analysis of the phenomenon should be conducted to have a picture of the relevance of the phenomenon and the users’ preferences and attitudes toward the use of digital stimuli related to grief. While conducting experimental studies might be delicate for ethical reasons, anonymous online surveys might shed light on the extension of the phenomenon and users’ opinions and preferences (Pizzoli et al., 2021).

Secondly, the issue of individual differences and related susceptibility to experiencing complicated grief and psychological pain and harm related to the exposure to digital stimuli representing the deceived should be carefully addressed. This would help create recommendations and guidelines, as well as the identification of fragile subjects. Risk factors of the specific event as well as pre-existing psychobiological features of the individuals might be assessed to predict the risk of complex grief (Shear & Kenworthy, 2012; Simon, 2012). Attachment style for instance is associated with the individual experience of grief (Shear & Shair, 2005), with people with anxious ambivalent attachment style suffering more intense pain (Fraley & Bonanno, 2004; Wayment & Vierthaler, 2011) and for more time (Fraley & Bonanno, 2004). Quality of existing relationships, financial status, and social isolation might be other risk factors to be considered (Norris & Murrell, 1990; Stroebe et al., 2007), as well as the conditions of the loss or the psychological pre-existing symptoms (for a complete overview see (Shear & Kenworthy, 2012).

Lastly, online guidelines, disclaimers, and information on relevant sites of psychological associations might be published, to allow an open sharing of risks and possibilities related to the topic for mental health professionals and laypeople. While in traditional therapies with grieving people, the therapist might ask the client to bring objects related to the deceased (as photos, audio or video clips) because it might help in the elaboration of grief (Worden, 2018), the psychological effects of AI digital interactions with the deceased are entirely unknown.

Lastly, the field of bereavement and psychological digital interventions need to be also carefully explored. Online digital support programs for grief began to be developed and some protocols for digital interventions to offer grief support have already been set up and studied. Specifically, protocols with psychoeducation, CBT, and compassion began to be applied for complicated grief (Tur et al., 2021) or bereaved caregivers (Uneno et al., 2022). Preliminary evidence on the efficacy of an 8-week web-based and virtual grief support program for widows yielded promising results on grief severity, loneliness, yearning, grief cognitions, perceived stress, and sleep quality (Knowles et al., 2017). The possibility of using or not audio clips in the treatments of bereavement should then be carefully considered within research protocol approved by ethical committees.

## Data Availability

Data availability is not applicable to this theoretical contribution as no datasets were generated or analysed in conducting the article.
